# Caesarean Delivery: A Narrative Review on the Choice of Neuraxially Administered Opioid and Its Implications for the Multimodal Peripartum Pain Concept

**DOI:** 10.3390/medicina60030358

**Published:** 2024-02-21

**Authors:** Mark Ulrich Gerbershagen, Hanaa Baagil

**Affiliations:** Department of Anaesthesiology, Teaching Hospital of the University Cologne, Hospital Cologne Holweide, University of Witten-Herdecke, Neufelder Str. 32, 51067 Cologne, Germany; baagilh@kliniken-koeln.de

**Keywords:** caesarean delivery, intrathecal opioid, neuraxial opioid, pain management, multimodal pain management, peripartum, local anesthesia, regional anesthesia

## Abstract

Nowadays, obstetrical anesthesia-related mortality is a very rare complication in industrialized countries. The recommended choice of intrathecal opioid for spinal anesthesia in the context of a multimodal peripartum pain management concept is discussed in this narrative review. Nowadays, there is a consensus that a perioperative multimodal pain concept should be used for caesarean delivery. This pain concept should include neuraxial opioids for spinal anesthesia, acetaminophen, NSAIDs, intravenous dexamethasone, and postoperative local or regional anesthetic procedures. Long-acting lipophobic opioids (diamorphine and morphine) have a significant analgesic advantage over short-acting lipophilic opioids (sufentanil and fentanyl). The risk of clinically relevant respiratory depression after neuraxial long-acting opioids is nowadays considered negligible, even if the data situation is weak in this regard. The question remains as to whether a pain concept that is ideally adapted to a neuraxial short-acting opioid shows benefit to a pain concept that is optimally adapted to neuraxial morphine. If long-acting opioids are used, the timing of each additional component of the multimodal analgesia strategy could ideally be adjusted to this longer duration of action.

## 1. Introduction

Most women worldwide experience childbirth at least once in their lives. Again, in many countries, the rate of caesarean sections in the total number of births has increased from 30 to 40% [1]. Thus, caesarean birth is the most frequently performed operation today before cataract surgery. The increase in the rate of caesarean sections is due to more generous medical indications on the one hand, and to the increasingly existing consensus in developed countries today that expectant mothers are allowed to decide on the modalities of the birth themselves in the sense of an “informed choice” in the event of an uncomplicated pregnancy and an expected uncomplicated birth, on the other hand. In many women, caesarean birth is associated with moderate-to-severe postoperative pain [2]. Any post-caesarean pain management regime must address the following points: the mothers should be mobilizable early since they are at significant risk for thromboembolic complications, they should be able to have early bonding, they should be able to take care of their newborns early, and they should be able to breastfeed. Consequently, pain management should be an essential part of the perioperative standard operating procedures. As mentioned, the evidence for individual analgesic measures—albeit carried out millions of times—is surprisingly low. 

The further goal of sufficient pain therapy is the prevention of long-term problems or complications. Above all, chronic pain after caesarean section should be mentioned here, which in turn is associated with a reduced “health related quality of life” and “anxiety symptoms”. The incidence of chronic pain after caesarean section is determined between 6.1% and 11.5%, depending on the definition of the chronic pain [3], and 9.6% of chronic pain patients after caesarean section complain of severe pain. As described, the caesarean section is a procedure that is carried out millions of times a year and occurs relatively early in a woman’s life, and the relevance of chronic pain after caesarean section should not be underestimated. Individual medical, psychological, and financial consequences for the mother but also for the national economy can be considerable. The predominant preoperative risk factor for postoperative chronic pain was the anamnesis of chronic pain, regardless of its location [4]. As for chronic postoperative pain after other surgeries, it can also be stated for chronic pain after caesarean section that the intensity as well as the duration of acute postoperative pain are the main postoperative predictors [5]. At this point, preexisting chronic pain is the main predictor of a higher pain intensity of acute postoperative pain [6]. It would be desirable to state that a specific anesthesiological or perioperative pain regimen would have a beneficial effect on the incidence of postoperative chronic pain after caesarean section. However, the data are not yet sufficient to answer this question. While this review does not focus on the pros and cons of caesarean section versus vaginal birth, it should be noted that the data suggest that caesarean section appears to be associated with less chronic pain than vaginal birth. In particular, the risk of “chronic pelvic pain” is significantly reduced compared to vaginal birth due to the lower pelvic trauma [7].

Neuraxial procedures, mostly spinal but also epidural anesthesia, have been preferred for some time compared to general anesthesia for caesarean birth [8,9]. To our knowledge, the current status of general anesthesia rates for elective cesarean delivery in most countries remains unknown. Nevertheless, it can be assumed that general anesthesia is used well below 5% of elective caesarean sections in developed countries [10,11]. In this context, spinal anesthesia has advantages in terms of onset time, simpler technical implementation, and cost-effectiveness. In addition, a significant benefit for the neuraxial procedures is the wakefulness of the parturient and, consecutively, a better birth experience for the expectant mother as well as for the father. The latter would not be present at birth in the case of a cesarean birth under general anesthesia. Spinal anesthesia has a very long tradition in obstetric anesthesia. This was used for labor pain for the first time in 1901 and one year later for the first time for caesarean delivery. Spinal anesthesia is now the most common anesthesia procedure for caesarean section in most countries [12,13,14,15]. During a spinal puncture, the dura mater is usually perforated with a fine, atraumatic cannula in the space between lumbar vertebral bodies 4 and 5 or 3 and 4. If there is a free flow of cerebrospinal fluid as well as the exclusion of blood via the spinal needle and the exclusion of paresthesia/dysesthesia, it can be assumed that the spinal needle is placed correctly. Subsequently, the drugs are administered intrathecally in a bolus, with repeated cerebrospinal fluid aspiration controls.

Through the intrathecal application of opioids, it is possible to reduce the dosage of the local anesthetic if the onset of action is similar to the administration of a long-acting local anesthetic alone (e.g., bupivacaine 0.5% hyperbaric). Furthermore, a similar or significantly longer duration of action after application can be achieved [14,16]. The reduction in dosage of the local anesthetic, in turn, should lead to a higher hemodynamic stability of the expectant mother during the caesarean section. On the other hand, intrathecal opioids can increase the likelihood of side-effects such as nausea, vomiting, pruritus, and respiratory depression [14,17,18,19]. The opioids sufentanil, fentanyl, pethidine, meperidine, diamorphine, and morphine have been described in studies for intrathecal use in caesarean delivery. The advantages and disadvantages of the individual opioids can be explained by differences in pharmacokinetics as well as pharmacodynamics, whereby lipid solubility is of very high importance [20,21]. A high lipophilicity of an opioid (e.g., sufentanil and fentanyl) leads to a relatively fast onset of action but also a shorter duration of action compared to opioids with a lower lipophilicity (e.g., diamorphine and morphine). In addition, the intrathecal dosage of the individual opioid has a significant influence on the onset as well as the duration of action [19]. When answering whether a short-acting lipophilic or a long-acting lipophobic neuraxial opioid is preferable for caesarean delivery, their respective embedding in a multimodal perioperative pain concept and the respective effect on safety aspects, acute post-caesarean pain, and chronic post-caesarean pain must be considered.

A modern post-caesarean pain management concept includes neuraxial techniques, opioids (neuraxial, oral, intravenous), systemic adjuncts, and local and regional anesthetic techniques. To anticipate this, there is currently no randomized controlled trial that would have compared a short-acting versus a long-acting neuraxial opioid embedded in a modern multimodal pain concept tailored to the pharmacokinetic of the individual opioid. The aim of this review is to present existing evidence and data and finally to hold out the prospect of what the potentially best integration of lipophilic short-acting as well as lipophobic long-acting opioids of the multimodal post-caesarean pain strategy could look like. These proposals would have to be tested in randomized controlled trials.

## 2. Materials and Methods

The primary objective of this narrative literature review was to systematically collect and summarize existing data on the impact of intrathecal opioids on safety aspects, pain perception, and peripartum pain concepts. The databases of PubMed, Cochrane Library, Medline, and Google Scholar were searched for the relevant articles from January 2013 to October 2023. The population search terms applied were the following: caesarean or caesarean section. The output from this search was combined with the terms intrathecal opioid or neuraxial opioid or safety or pain management or regional anesthesia or local anesthesia. Articles that provided no abstract were excluded. Inclusion criteria were established based on the primary endpoint of low post-caesarean pain associated with intrathecal opioid or peripartum pain management, with the aim of comprising a broad and impartial selection of relevant research studies.

## 3. Results

### 3.1. Neuroaxial Anaesthesia and Safety Aspects

The two main safety arguments for the use of neuraxial blockades are the avoidance of difficult airway situations [22,23] and aspiration as well as the reduced potential for neonatal compromise [24]. Mainly by switching from general anesthesia to neuraxial blockade, especially spinal anesthesia—as the standard anesthesia procedure for caesarean section—the anesthesia-related mortality of the mothers was significantly reduced. Between 1985 and 1987, anesthesia-related maternal deaths occurred in 6 out of 100,000 maternities in the United Kingdom. Anesthesia-related maternal deaths between 2014 and 2019 could be reduced to 1 per 100,000 maternities in the UK [15]. An even further reduction in anesthesia-related maternal mortality was monitored in the UK between 2019 and 2021: 0.05/100,000 maternities [25]. At this point, the reader should be reminded of the difference between anesthesia-related and anesthesia-associated mortality. Anesthesia-related mortality: the death of the patient is directly caused by anesthesia or anesthesia procedures in the narrower sense (death due to airway mismanagement, toxicity of local anesthetics, etc.). Anesthesia-associated mortality: patients die during anesthesia (e.g., death due to hemorrhage during anesthesia, amniotic fluid embolism during anesthesia, pulmonary artery embolism during anesthesia). Logically, anesthesia-associated maternal mortality is significantly higher than anesthesia-related maternal mortality. However, the data for anesthesia-associated maternal mortality does not exist in the UK data, for example, because these maternal deaths are subsumed under direct obstetric deaths [25]. Even though this reduction in maternal death is already a great success, there is an effort to further reduce anesthesia-related maternal death and maternal complications. It should be noted that due to the frequent use of regional anesthesia (spinal and epidural anesthesia) to facilitate vaginal birth as well as for caesarean birth compared to general anesthesia, it is also logical that, today, the rare event of maternal death in the majority of cases is associated with spinal or epidural anesthesia. Clearly, there is no current statistical difference between neuraxial blockade and general anesthesia in elective cesarean delivery for maternal mortality or cardiac arrest [11,26]. However, in a retrospective study, it was demonstrated that avoidable general anesthetics for elective caesarean delivery were associated with increased risk of anesthesia complications, increased severe complications, increased surgical site infection, and increased venous thromboembolism compared to neuraxial procedures [11]. Keeping this in mind, the goal must be to further reduce complications associated with spinal and epidural anesthesia. One of the complications is respiratory depression, which can be reduced by consequently optimizing the monitoring of vital parameters of the mothers after birth. In a British NICE guideline from 2021, the following review question was investigated: “What post-operative monitoring is required for women who have received intrathecal or epidural opioids at the time of caesarean birth, to identify or prevent potential complications (including the duration, frequency and features to be monitored)?” [15]. With regard to clinical evidence, the Guideline Committee concludes: “A systematic review of the clinical literature was conducted but no studies were identified which were applicable to this review question.” While the old NICE guidelines still recommended maternal vital signs to be monitored at least hourly for at least 12 h and for intrathecal morphine at least 24 h after intrathecal diamorphine application, the current NICE guidelines from 2021 pronounce a practical approach: “After caesarean birth under a spinal or epidural anaesthetic, a healtcare professional should carry out continuous one-to-one observation of the woman until she is haemodynamically stable”.

Since diamorphine intrathecal is mainly used for caesarean birth in the UK today and morphine is hardly used, intrathecal morphine is no longer explicitly mentioned in the NICE guidelines of 2021. Due to the higher lipid solubility of diamorphine compared to morphine, the likelihood of delayed onset respiratory depression also appears to be decreased [27,28]. Only in a patient with respiratory risk factors (obesity, obstructive sleep apnea syndrome) after administration of spinal or epidural diamorphine is post-caesarean vital monitoring (continuous pulse oximetry monitoring, and hourly monitoring of respiratory rate, heart rate, blood pressure, temperature, pain, and sedation) stipulated for at least 12 h. After that, local protocols should be used [15]. The current NICE guidelines appear to be a good compromise between safety aspects and practicability. 

### 3.2. Comparison of Short-Acting versus Long-Acting Neuroaxial Opioids

Although intrathecal opioids are used daily worldwide in combination with local anesthetics, there is no clear study situation as to which opioid is ideal for caesarean delivery. In the last 25 years, five systematic reviews have dealt with this question in the broadest sense, as well as several randomized controlled trials [29,30,31,32,33,34,35,36,37]. The first systematic review found that, in a relatively small total number of patients, intrathecal fentanyl and sufentanil have no advantage over the administration of local anesthetics alone. On the other hand, morphine as an adjacent has been shown to have an extended time to the first postoperative analgetic requirement as well as reduced pain scores [38]. Another systematic review in 2016 dealt only with the question of intrathecal morphine dosing. The morphine dosages were divided into low- (50 to 100 μg) and high-dosage groups (100 to 250 μg), whereby the cut-off point of 100 μg morphine is to be interpreted as arbitrary. The higher morphine dosage was associated with a longer analgetic effect, but also with a higher probability of occurrence of pruritus, nausea, and vomiting [39]. Another meta-analysis addressed only the comparison of diamorphine and morphine on postoperative respiratory depression. Thus, there was no case of either respiratory depression or low respiratory rates with intrathecal morphine or intrathecal diamorphine [27]. The randomized controlled trials of recent years confirm the safe use with good postoperative pain reduction in the neuraxial morphine with optimization of the dosage [33,34,35,36,37,39]. The most comprehensive systematic review to date on the topic of intrathecal opioid and elective caesarean section was published in 2021 [14]. The effect of five opioids (sufentanil, fentanyl, meperidine, diamorphine, and morphine) and the combination of fentanyl and morphine on seven clinical outcomes (duration of complete analgesia with pain VAS = 0, length of time to pain ≥ 4, time to the first analgesic use, 24 h opioid consumption) was analyzed. All intrathecally administered opioids significantly reduced the time of complete analgesia as well as time until VAS ≥ 4 to placebo. However, no lipophilic opioid (sufentanil, fentanyl, and meperidine) reduced cumulative 24 h opioid consumption or delayed time to the first analgesic use compared to placebo [29,30,31,32]. Relative to lipophilic substances, the 24 h consumption of further opioids after neuraxial diamorphine and morphine was significantly lower, with no difference between diamorphine and morphine. Furthermore, morphine (mean 660 min, 95% CrI: 510–810 min) as well as diamorphine (mean 230 min, 95% CrI: 26–440 min) were associated with a longer time to the first analgetic use compared to placebo.

The most feared side-effect of intrathecally administered opioids is respiratory depression. In 83% of the studies included in this meta-analysis, neither in placebo nor in opioid groups was respiratory depression detected. Nevertheless, it is noteworthy that, although very rare, there was a significant increase in the incidence of respiratory depression for both sufentanil and morphine. In the case of sufentanil, however, respiratory depression seems to occur relatively early, but in the case of morphine, it is severely delayed. From a statistical point of view, however, the authors interpret that “the results on respiratory depression obtained here are inconclusive” [14]. All neuraxial opioids, except for diamorphine, were associated with pruritus. Regarding low-certainty evidence, no reliable statement can be made about the likelihood of nausea and vomiting occurring [14]. A recent study shows that the incidence of nausea was 20.2% after intrathecal morphine and 7.6% after intrathecal fentanyl. The difference was even more evident regarding vomiting: vomiting occurred in 12.8% of mothers after intrathecal morphine, whereas it was not reported after intrathecal fentanyl. In both groups, intraoperative prophylaxis against nausea with dexamethasone and granisetron was performed intravenously [40]. The authors of the meta-analysis conclude that neuraxial morphine adjacent to a local anesthetic is the most appropriate substance from an analgesic point of view. Unfortunately, since data and evidence on side-effects appear to be limited, this most comprehensive systemic review to date also concludes: “We cannot ascertain confidently which opioid is best for addition to local anesthetic for caesarean section” [14]. 

### 3.3. Local and Regional Anesthesia Procedures

In the last 15 years, abdominal wall fascial plane blocks have become fashionable for a wide variety of surgical procedures. Only the widespread introduction of ultrasound in anesthesia departments and the increasing expertise with ultrasound have made these regional techniques possible. While there are a variety of approaches for abdominal wall blocks, at this point, the focus is on the three blocks recommended by the PROSEPT initiative for caesarean birth: transversus abdominal plane (TAP) block, quadratus lumborum block (QLB), and erector spinae plane block [41].

In contrast to the TAP block, the deposition of local anesthetic adjacent to the quadratus lumborum muscle addresses not only the somatic pain associated with a Pfannenstiel incision but may also address the visceral component of the pain after caesarean birth. The visceral effect is explained by the potential spread of local anesthetics proximally to the paravertebral space [42,43]. In studies on volunteers, it was shown that an anterior quadratus lumborum block with 20, 30, and 40 mL of 0.375% ropivacaine causes a cold sensation block duration of 21, 24, and 24 h (median time) [44]. Another study was able to demonstrate a spread of a median of three dermatomes for both the quadratus lumborum block and the transversus abdominal plane block after 20 mL of 0.375% levobupivacaine, whose number of dermatomes with a loss of cold sensation and pin-prick sensation slowly decreased after 6 h [45]. Patients after gynecological procedures under general anesthesia (not caesarean section) and quadratus lumborum block felt pain at rest after 6 h with VAS 2.3 ± 2.3 and pain while coughing after 6 h with VAS 4.1 ± 2.3. The cumulative i.v. fentanyl (patient-controlled analgesia) consumption was 73 ± 35 μg after 6 h postoperatively. The data were similar for the same surgical indication after general anesthesia and transversus abdominal plane block, with pain at rest after 6 h with VAS 3.2 ± 2.2 and pain while coughing after 6 h with 4.9 ± 2.8, respectively, as well as cumulative fentanyl consumption with 67 ± 32 μg after 6 h [45]. After both blockade techniques, in the majority of cases, there was a sensory block in the dermatomes Th 11 and 12; with regard to other dermatomes, there was a high variability. In particular, the mean pain after coughing after 6 h may indicate that the somatic analgesic effect decreases earlier than expected after these two blocks. This could also be the main reason why a directly postoperatively performed quadratus lumborum block does not enhance analgesic outcomes when combined with or compared with spinal morphine [46]. This shows similar rest pain scores and analgesic consumption during the first 24 h postoperatively. On the other hand, studies using lipophilic opioids (sufentanil or fentanyl) in combination with a local anesthetic provide strong evidence that a quadratus lumborum block applied directly postoperatively exerts a positive analgesic effect: improved acute rest pain control at 4 to 6 h and analgesic consumption during the first 24 h postoperatively, as well as a clinically important improvement in overall rest pain over the 48 h interval [47,48].

On the other hand, infiltration of the wound edges with local anesthetics by the surgeon can be considered [49,50]. The advantage compared to fascial plane blocks is that local wound infiltration is an easier procedure to perform and does not require ultrasound knowledge. In fact, studies show that local wound infiltration of the Pfannenstiel incision is comparable with fascia blocks after caesarean section [51,52]. For example, a meta-analysis summarizes that 2 and 12 h postoperatively, there was no difference between local wound infiltration and TAP block in terms of pain score or further intravenous morphine requirements of the young mothers [53]. A prospective RCT compared the effect of local wound infiltration with posteromedial quadratus lumborum block after the intrathecal administration of hyperbaric bupivacaine and fentanyl (20 μg) [54]. In this study, the same multimodal pain concept was implemented for both groups: intravenous paracetamol 1 g 6 hourly and metamizole 2.5 g at 12-hourly intervals, both starting 2 h after surgery. In addition, intravenous Neodolpasse 250 mL (diclofenac 75 mg, orphenadrine 30 mg) was administered 4 h postoperatively. The multimodal pain concept applied in both the wound infiltration group and the quadratus lumborum group can be described as very good. In both groups, intravenous piritramide was consumed to a small extent within 24 h postoperatively (wound infiltration group median 2.2 mg piritramide and quadratus lumborum group 1.5 mg piritramide), with a median pain intensity of 2 reported within 48 h postoperatively in both groups. In the period from 24 to 48 h, almost no further piritramid was retrieved from the patients in both groups. The time to the first analgesic request was 11 h (median) for the QLB group compared to 7 h (median) for the wound infiltration group. It is possible that the slight advantage of QLB can be attributed to the fact that this technique can reduce visceral pain to a certain extent. Another study showed a benefit of subcutaneous wound infiltration with ketamines compared to bupivacaine [55]. However, this observation would have to be confirmed by further studies.

### 3.4. Dexamethasone

There are now numerous studies that focus on the perioperative application of dexamethasone in a wide variety of types of surgery. The aim is to prolong analgesia but also to reduce side-effects of intrathecal opioid, like nausea [56], and vomiting and itching [57]. The anti-inflammatory as well as analgesic effect of dexamethasone is based on the inhibitory effect on the phospholipase enzyme and consecutively on the cyclooxygenase and lipoxygenase pathway.

In caesarean birth, the intravenous administration of 8 mg of dexamethasone is recommended after delivery [58,59,60]. The advantage of dexamethasone administration is particularly evident when administered after using neuraxial short-acting lipophilic opioids. A study could show lower pain severity 4 to 5 h postoperatively and a significantly later time to the first rescue of analgesia compared to a group without dexamethasone administration [58]. On the other hand, the intraoperative administration of intravenous dexamethasone after long-acting neuraxial opioids (morphine, diamorphine) shows a lesser statistical advantage to no administration of dexamethasone [58,61,62]. The common side-effects of intrathecal morphine such as pruritus, which has been reported to occur between 36% and 60%, could be reduced with intravenous dexamethasone. However, the combination of 8 mg of dexamethasone and 4 mg of ondansetron (13.5% pruritus) showed no superiority to the monotherapy of 4 mg of ondansetron (11.5% pruritus) after intrathecal morphine [57]. It should be mentioned that studies have dealt with the question of whether intrathecal dexamethasone or an adjacent in a facial plane block [63] prolongs pain reduction. Even if a positive pain-reducing effect can be demonstrated, the advantage compared to intravenous dexamethasone remains questionable with increased concerns about safety issues.

## 4. Discussion

The use of an intrathecal opioid for cesarean section has been characterized by tradition rather than evidence in recent decades. This tradition can refer to a hospital, a region, or even a nation. Anesthesiologists in the USA and Great Britain have had a lot of good experience with intrathecal morphine as well as diamorphine. Accordingly, the PROSPECT Working Group of the European Society of Regional Anaesthesia and Pain Management confirmed the existing evidence and recommended the use of intrathecal long-acting opioids (e.g., morphine 50–100 μg or diamorphine up to 300 μg) at a European level [41]. This recommendation was reaffirmed in the latest update of the PROSPECT Working Group [64]. On the other hand, Seki et al. clearly stated in their most comprehensive systemic review on this topic to date in 2021 that as the data and evidence on side-effects appear to be limited, it is still unclear which opioid is best used as an adjunct to local anesthesia for caesarean section [14]. Appropriately, the PROSPECT Working Group’s recommendation to prefer long-acting opioids should be interpreted as the best possible compromise between evidence and best practice [41,64].

In nations, such as Germany, concerns about the potential side-effects in the past have led national professional societies to define such high standards for postoperative monitoring after intrathecal long-acting opioids that intrathecal morphine or diamorphine was de facto not applicable in the past. Accordingly, almost exclusively short-acting opioids were used intrathecally. Additionally, many years of good experience, especially with sufentanil, and the fast-acting time after application, led to the rare use of diamorphine and morphine in Germany. It is certainly correct to state that a large number of anesthesiologists in Germany have no experience with the intrathecal application of long-acting opioids. The use of sufentanil is an established off-label use, which is even explicitly recommended in the S1 guideline “Obstetric analgesia and anesthesia” [13]. However, the current German S3 guideline “Treatment of acute perioperative and post-traumatic pain” also recommends intrathecal lipophilic opioids (sufentanil or fentanyl) or morphine in doses of 50–100 μg [65].

Nevertheless, the following question needs to be clarified scientifically: Is there any evidence that a peripartum pain concept optimally adapted to a long acting opioid is better than a peripartum pain concept optimally adapted to a short-acting opioid? The answer is no. To date, there is no evidence to support this statement. When creating an optimal peri-cesarean pain strategy, the different duration of action of neuraxial opioids must be considered: It is to be expected that young mothers will feel pain with a VAS score of 4 on average 180 min after intrathecal application of a lipophilic short-acting opioid (e.g., fentanyl, sufentanil). With a caesarian section lasting approximately 60 min, the pain will occur 120 min postoperatively [16]. On average, it is to be expected that the young mothers will ask for the first pain medication 660 min (95% Crl: 510–810 min) after intrathecal morphine in combination with a local anesthetic. However, the analgesic effect of neuraxial diamorphine is significantly shorter: the mean time the first analgesic was used was described after 230 min (95% Crl: 26–440 min).

Ideally, the pain concept optimally adapted to a short-acting opioid should be defined with corresponding evidence, before it is compared to the optimal pain concept for morphine and diamorphine. Optimal study designs should evaluate preoperative items such as pain management strategies, any chronic pain, depression, and anxiety. Postoperatively, in addition to the direct pain-related items, such as pain onset, pain intensity, duration of pain, the mothers’ satisfaction with the respective multimodal pain management, breastfeeding, and bonding should be evaluated [66]. The exposure of the mother to potential side-effects such as nausea and vomiting as well as pruritus are further important parameters. Additionally, follow-up interviews with the mother are important in order to detect medium- and long-term differences in the individual pain concepts and to evaluate the incidences of any new chronic pain. 

For intrathecal morphine and the optimal multimodal postoperative pain therapy, one finding emerges: If a pain therapy procedure enables almost 100 percent freedom from pain for a defined period, then another analgesic procedure, which in all likelihood only unfolds its effect during this period, will not provide a significant additional analgesic benefit. This assumption was confirmed by studies regarding intrathecal morphine and additional postoperative fascial blocks, local anesthesia procedures, and dexamethasone [50] applied immediately after completion of the caesarean section [42,46,67,68]. Statements such as the following are therefore misleading and not generalizable: “Moderate quality evidence suggests that quadratus lumborum block does not enhance analgesic outcomes when combined with or compared with spinal morphine” [46]. However, it is true that an additional analgesic benefit would only be expected if further regional anesthesia procedures were applied towards the end of the statistical analgesic duration of intrathecal morphine. An ideal time for an additional local or regional anesthetic procedure after morphine i.t. would certainly be 4 to 8 h after caesarean birth in the statistically last quarter of the analgesic duration of action. However, since the young mothers are usually already on the normal ward at this time and are no longer in anesthesiologic care, the logistical effort for wound infiltration or an ultrasound-guided regional anesthetic technique is much higher, but not impossible. Future studies would have to show whether postoperative pain concepts that take these considerations into account have an analgesic benefit as well as appear practicable.

It can be assumed that the optimal pain concepts tailored to the respective intrathecal opioid will nevertheless continue to be very similar in the future. Today’s recommendations are preoperative oral paracetamol, intra-operative after delivery intravenous paracetamol, intravenous non-steroidal anti-inflammatory drugs, intravenous dexamethasone, wound infiltration or facial plane blocks if neuraxial morphine is not used, postoperative oral or intravenous paracetamol, oral or intravenous non-steroidal anti-inflammatory drugs, and opioids for rescue [41,64]. Future differences in pain concepts could arise mainly with regard to timing and the standardized repetition of drug administration. In the case of the use of neuraxial sufentanil or fentanyl, the standardized pain-level-adapted oral administration of opioids could be a more important component than in a pain concept for neuraxial morphine [69]. For the ideal pain concept adapted to neuraxial morphine, the standardized time points of further analgetic administration could be delayed. As already described, a local or regional anesthesia procedure 4–8 h after delivery could be useful.

## 5. Conclusions

Nowadays, anesthesia-related mortality is a very rare complication in industrialized countries, mainly because spinal anesthesia has become the standard anesthesia procedure for caesarean birth. Nowadays, there is a consensus that a perioperative multimodal pain concept should be used for caesarean delivery. This pain concept should include neuraxial opioids for spinal anesthesia, paracetamol, non-steroidal anti-inflammatory drugs, intravenous dexamethasone, and postoperative local or regional anesthetic procedures. Long-acting lipophobic opioids (diamorphine and morphine) have a significant analgesic advantage over short-acting lipophilic opioids (sufentanil and fentanyl). The risk of clinically relevant respiratory depression after neuraxial long-acting opioids is nowadays considered negligible, even if the data situation is weak in this regard. 

The question remains as to whether a pain concept that is ideally adapted to a neuraxial short-acting opioid shows benefit to a pain concept that is optimally adapted to neuraxial morphine. If long-acting opioids are used, the timing of each additional component of the multimodal analgesia strategy could ideally be adjusted to this longer duration of action.

## References

[1] Antoine C., Young B.K. (2020). Cesarean section one hundred years 1920–2020: The Good, the Bad and the Ugly. J. Perinat. Med..

[2] Gamez B.H., Habib A.S. (2018). Predicting severity of acute pain after Cesarean delivery: A narrative review. Anesth. Analg..

[3] Weibel S., Neubert K., Jelting Y., Meissner W., Wöckel A., Roewer N., Kranke P. (2016). Incidence and severity of chronic pain after caesarean section: A systematic review with meta-analysis. Eur. J. Anaesthesiol..

[4] Schug S.A., Bruce J. (2017). Risk stratification for the development of chronic postsurgical pain. Pain Rep..

[5] Fletcher D., Stamer U.M., Pogatzki-Zahn E., Zaslansky R., Tanase N.V., Perruchoud C., Kranke P., Komann M., Lehman T., Meissner W. (2015). euCPSP group for the Clinical Trial Network group of the European Society of Anaesthesiology. Chronic postsurgical pain in Europe: An observational study. Eur. J. Anaesthesiol..

[6] Lavande’Homme P. (2019). Postpartum chronic pain. Minerva Anestesiol..

[7] Bijl R.C., Freeman L.M., Weijenborg P.T., Middeldorp J.M., Dahan A., van Dorp E.L. (2016). A retrospective study on persistent pain after childbirth in the Netherlands. J. Pain Res..

[8] Apfelbaum J.L., Hawkins J.L., Agarkar M., Bucklin B.A., Connis R.T., Gambling D.R., Mhyre J., Nickinovich D.G., Sherman H., Tsen L.C. (2016). Practice guidelines for obstetric anesthesia: An updated report by the American Society of Anesthesiologists Task Force onObstetric Anesthesia and the Society for Obstetric Anesthesia and Perinatology. Anesthesiology.

[9] Committee on Practice Bulletins—Obstetrics (2017). Practice bulletin No. 177: Obstetric analgesia and anesthesia. Obstet Gynecol..

[10] Yonekura H., Mazda Y., Noguchi S., Tsunobuchi H., Shimaoka M. (2022). Current epidemiology of the general anesthesia practice for cesarean delivery using a nationwide claims database in Japan: A descriptive study. J. Clin. Med..

[11] Guglielminotti J., Landau R., Li G. (2019). Adverse events and factors associated with potentially avoidable use of general anesthesia in cesarean deliveries. Anesthesiology.

[12] D’Angelo R., Smiley R.M., Riley E.T., Segal S. (2014). Serious complications related to obstetric anesthesia: The serious complication repository project of the Society for Obstetric Anesthesia and Perinatology. Anesthesiology.

[13] Bremerich D., Annecke T., Chappell D., Hanß R., Kaufner L., Kehl F., Kranke P., Girad T., Gogarten W., Greve S. Die Geburtshilfliche Analgesie und Anästhesie. S1-Leitlinie der Deutschen Gesellschaft für Anästhesiologie und Intensivmedizin in Zusammenarbeit mit der Deutschen Gesellschaft für Gynäkologie. 2020; AWMF-Leitlinie. https://register.awmf.org/assets/guidelines/001-038l_S1_Die-geburtshilfliche-Analgesie-und-Anaesthesie_2020-03.pdf.

[14] Seki H., Shiga T., Mihara T., Hoshijima H., Hosokawa Y., Hyuga S., Fujita T., Koshika K., Okada R., Kurose H. (2021). Effects of intrathecal opioids on cesarean section: A systematic review and Bayesian network meta-analysis of randomized controlled trials. J. Anesth..

[15] (2021). NICE Guideline NG192. Caesarean Birth. https://www.nice.org.uk/guidance/NG192.

[16] Vyas N., Sahu D.K., Parampill R. (2010). Comparative study of intrathecal sufentanil bupivacaine versus intrathecal bupivacaine in patients undergoing elective cesarean section. J. Anaesthesiol. Clin. Pharmacol..

[17] Bremerich D. (2019). Pain therapy after C-section. Anästh. Intensivmed..

[18] Chen X., Qian X., Fu F., Fu H., Bein B. (2010). Intrathecal sufentanil decreases the median effective dose (ED50) of intrathecal hyperbaric ropivacaine of caesarean delivery. Acta Anaesthesiol. Scand..

[19] Sultan P., Gutierrez M.C., Carvalho B. (2011). Neuroaxial morphine and respiratory depression: Finding the right balance. Drugs..

[20] Agrawal A., Asthana V., Sharma J.P., Gupta V. (2016). Efficacy of lipophilic vs lipophobic opioids in addition to hyperbaric bupivacaine for patients undergoing lower segment caesarean section. Anesth. Essays. Res..

[21] Staikou C., Paraskeva A. (2014). The effects of intrathecal and systemic adjuvants on subarachnoid block. Minerva Anesthesiol..

[22] Kinsella S.M., Winton A.L., Mushambi M.C., Ramaswamy K., Swales H., Quinn A.C., Popat M. (2015). Failed tracheal intubation during obstetric general anaesthesia: A literature review. Int. J. Obstet. Anesth..

[23] Quinn A.C., Milne D., Columb M., Gorton H., Knight M. (2013). Failed tracheal intubation in obstetric anaesthesia: 2 yr national case-control study in the UK. Br. J. Anaesth..

[24] Ong B.Y., Cohen M.M., Palahniuk R.J. (1989). Anesthesia for cesarean section–effects on neonates. Anesth. Analg..

[25] Maternal Mortality 2019–2021. https://www.npeu.ox.ac.uk/mbrrace-uk/data-brief/maternal-mortality-2019-2021.

[26] Hawkins J.L. (2003). Anesthesia-related maternal mortality. Clin. Obstet. Gynecol..

[27] Sharawi N., Carvalho B., Habib A.S., Blake L., Mhyre J.M., Sultan P. (2018). A systematic review evaluating neuraxial morphine and diamorphine-associated respiratory depression after cesarean delivery. Obstet. Anesthesiol..

[28] Amstrong S., Fernando R. (2016). Side-effects and efficacy of neuraxial opioids in pregnant patients at delivery: A comprehensive review. Drug Safety.

[29] Abdollahpour A., Azadi R., Bandari R., Mirmohammadkhani M. (2015). Effects of adding midazolam and sufentanil to intrathecal bupivacaine on analgesia quality and postoperative complications in elective cesarean section. Anesthesiol. Pain Med..

[30] Ali W.A., Mohammed M., Abdelraheim A.R. (2020). Effect of intrathecal fentanyl on the incidence, severity, and duration of postdural puncture headache in parturients undergoing caesarean section: A randomised controlled trial. Indian J. Anaesth..

[31] Bhattacharjee A., Singh N.R., Singh S.S., Debbrama B., Debbarma P., Singh T.H. (2015). A comparative study of intrathecal clonidine and fentanyl along with bupivacaine in spinal anesthesia for caesarean section. J. Med. Soc..

[32] Bindra T.K., Kumar P., Jindal G. (2018). Postoperative analgesia with intrathecal nalbuphine versus intrathecal fentanyl in cesarean section: A double-blind randomized comparative study. Anesth. Essays Res..

[33] Berger J.S., Gonzalez A., Hopkins A., Alshaeri T., Jeon D., Wang S., Amdur R.L., Smiley R. (2016). Dose-response of intrathecal mor-phine when administered with intravenous ketorolac for post-cesarean analgesia: A two-center, prospective, randomized, blinded trial. Int. J. Obstet. Anesth..

[34] Booth J.L., Harris L.C., Eisenach J.C., Pan P.H. (2016). A randomized controlled trial comparing two multimodal analgesic tech-niques in patients predicted to have severe pain after cesarean delivery. Anesth. Analg..

[35] Carvalho B., Mirza F., Flood P. (2017). Patient choice compared with no choice of intrathecal morphine dose for caesarean analgesia: A randomized clinical trial. Br. J. Anaesth..

[36] Carvalho B., Sutton C.D., Kowalczyk J.J., Flood P.D. (2019). Impact of patient choice for different analgesic protocols on opioid consumption: A randomized prospective trial. Reg. Anesth. Pain Med..

[37] Sharpe E.E., Molitor R.J., Arendt K.W., Torbenson V.E., Olsen D.A., Johnson R.L., Schroeder D.R., Jacob A.K., Niesen A.D., Sviggum H.P. (2020). Intrathecal morphine versus intrathecal hydromorphone for analgesia after caesarean delivery. Anesthesiology.

[38] Dahl J.B., Jeppesen I.S., Jørgensen H., Wetterslev J., Møiniche S. (1999). Intraoperative and postoperative analgesic efficacy and adverse effects of intrathecal opioids in patients undergoing cesarean section with spinal anesthesia: A qualitative and quantitative systematic review of randomized controlled trials. Anesthesiology.

[39] Sultan P., Halpern S.H., Pushpanathan E., Patel S., Carvalho B. (2016). The effect of intrathecal morphine dose on outcomes after elective cesarean delivery: A meta-analysis. Anesth. Analg..

[40] Botea M.O., Diana Lungeanu D., Petrica A., Sandor M.I., Huniadi A.C., Barsac C., Marza A.M., Moisa R.C., Maghiar L., RaluBotea R.M. (2023). Perioperative analgesia and patients’ satisfaction in spinal anesthesia for cesarean section: Fentanyl versus morphine. J. Clin. Med..

[41] Roofthooft E., Joshi G.P., Rawal N., Van de Velde M., on behalf of the PROSPECT Working Group of the European Society of Regional Anaesthesia and Pain Therapy and supported by the Obstetric Anaesthetists Association (2022). PROSPECT guideline for elective caesarean section: Update systematic review and procedure-specific postoperative pain management recommendations. Anaesthesia.

[42] Tamura T., Yokota S., Ito S., Shibata Y., Nishiwaki K. (2019). Local anesthetic spread into the paravertebral space with two types of quadratus lumborum blocks: A crossover volunteer study. J. Anesth..

[43] Blanco R. (2016). The mechanism of the quadratus lumborum block: A peripheral sympathetic field block?. Br. J. Anaesth..

[44] Shao L., Luo X., Ye Y., Liu L., Cai Y., Xia Y., Papadimos T.J., Wang Q., Pan L. (2022). Anterior Quadratus Lumborum block area comparison in the three different volumes of ropivacaine: A double-blind, randomized controlled trial in healthy volunteers. BMC Anesthesiol..

[45] Aoyama Y., Sakura S., Abe S., Wada M., Saito Y. (2020). Analgesic effects and distribution of cutaneous sensory blockade of quadratus lumborum block type 2 and posterior transversus abdominis plane block: An observational comparative study. Korean J. Anesthesiol..

[46] Hussain N., Brull R., Weaver T., Zhou M., Essandoh M., Abdallah F.W. (2021). Postoperative analgesic effectiveness of quadratus lumborum block for cesarean delivery under spinal anesthesia. Anesthesiology.

[47] Hansen C.K., Dam MSteingrimsdottir G.E., Laier G.H., Lebech M., Poulsen T.D., Chan V.W.S., Wolmarans M., Bendtsen T.F., Børglum J. (2019). Ultrasound-guided transmuscular quadratus lumborum block for elective cesarean section significantly reduces postoperative opioid consumption and prolongs time to first opioid request: A double-blind randomized trial. Reg. Anesth. Pain Med..

[48] Krohg A., Ullensvang K., Rosseland L.A., Langesæter E., Sauter A.R. (2018). The analgesic effect of ultrasound-guided quadratus lumborum block after cesarean delivery: A randomized clinical trial. Anesth. Analg..

[49] Gabriel R.A., Burton B.N., Curran B.P., Urman R.D. (2021). Regional anesthesia abdominal blocks and local infiltration after cesarean delivery: Review of current evidence. Curr. Pain Headache Rep..

[50] Singh N.P., Monks D., Makkar J.K., Palanisamy A., Sultan P., Singh P.M. (2022). Efficacy of regional blocks or local anaesthetic infiltration for analgesia after caesarean delivery: A network meta-analysis of randomised controlled trials. Anaesthesia.

[51] Telnes A., Skogvoll E., Lonnee H. (2015). Transversus abdominis plane block vs. Wound infiltration in caesarean section: A randomised controlled trial. Acta Anaesthesiol. Scand..

[52] Tawfik M.M., Mohamed Y.M., Elbadraw R.E. (2017). Transversus abdominis plane block versus wound infiltration for analgesia after cesarean delivery: A randomized controlled trial. Anesth. Analg..

[53] Grape S., Kirkham K.R., Albrecht E. (2022). Transversus abdominis plane block versus local anaesthetic wound infiltration for analgesia after caesarean section. Eur. J. Anaesthesiol..

[54] Stopar-Pintaric T., Blajic I., Visic U., Znider M., Plesnicar A., Vlassakov K., Lucovnik M. (2021). Posteromedial quadratus lumborum block versus wound infiltration after caesarean section. Eur. J. Anaesthesiol..

[55] Aksoy H., Gökahmetoglu G., Ak M., Aksoy Ü. (2023). Subcutaneous wound infiltration of ketamine is superior to bupivacaine in terms of pain perception and opioid consumption after cesarean section: A double-blinded randomized placebo-controlled clinical trial. Eur. Rev. Med. Pharmacol. Sci..

[56] Bozorgmanesh M., Valibeik S., Shokrpour M., Maktabi M., Kamali A. (2022). Effect of combined paracetamol and dexmethasone vs. paracetamol on postoperative nausea vomiting after cesarean section. J. Perinat. Med..

[57] Ankouni T., Kanawati S., Khatib R.E., Hassan J.E., Itani S.E., Rajab O., Naja Z. (2021). Ondansetron versus ondansetron with dexamethasone to prevent intrathecal-morphine pruritus for caesarean patients: Randomised double-blind trial. J. Obstet. Gynaecol..

[58] Singh N.P., Makkar J.K., Yadav N., Goudra B.G., Singh P.M. (2022). The analgesic efficiency of intravenous dexamethasone for postcaesarean pain. A systematic review with meta-analysis and trial sequential analysis. Eur. J. Anaesthesiol..

[59] Kiasari A.Z., Aghaei N., Aezzi G., Alipour A., Ghavibonyeh K. (2022). Effects of intrathecal and intravenous dexamethasone on complications associated with intrathecal morphine after cesarean section: A comparative study. J. Edu. Health Promot..

[60] Maged A.M., Deeb W.S., Elbaradie S., Elzayat A.R., Metwally A.A., Hamed M., Shaker A. (2018). Comparison of local and intravenous dexamethasone on post operative pain and recovery after caesarean section. A randomized controlled trial. Taiwan J. Obstet. Gynecol..

[61] Cardoso M.M., Leite A.O., Santos E.A., Gozzani J.L., Mathias L.A.S.T. (2013). Effect of dexamethasone on prevention of postoperative nausea, vomiting and pain after caesarean section: A randomized, placebo-controlled, double-blind trial. Eur. J. Anaesthesiol..

[62] Ituk U., Thenuwara K. (2018). The effect of a single intraoperative dose of intravenous dexamethasone 8 mg on postcesarean delivery analgesia: A randomized controlled trial. Int. J. Obstet. Anesth..

[63] Aga A., Abrar M., Ashebir Z., Seifu A., Zewdu D., Teshome D. (2021). The use of perineural dexamethasone and transverse abdominal plane block for postoperative analgesia in cesarean section operatins under spinal anesthesia: An observational study. BMC Anesthesiol..

[64] Roofthooft E., Joshi G.P., Rawal N., Van de Velde M. (2023). PROSPECT guideline for elective caesarean section: An update. Anaesthesia.

[65] AWMF S3-Leitlinie “Behandlung Akuter Perioperativer und Posttraumatischer Schmerzen”. https://register.awmf.org/assets/guidelines/001-025l_S3_Behandlung-akuter-perioperativer-posttraumatischer-Schmerzen_2022-11.pdf.

[66] La Via L., Minardi C., Brancati S., Valenti S., Messina S., Garofalo E., Bruni A., Murabito P., Bernardini R., Sanfilippo F. (2023). Fentanyl vs morphine as adjuvant to spinal anesthesia for caesarean section: An observational study. Euromediterranean Biomed. J..

[67] Irwin R., Stanescu S., Buzaianu C., Rademan M., Roddy J., Gormley C., Tan T. (2020). Quadratus lumborum block for analgesia after caesarean section: A randomised controlled trial. Anaesthesia.

[68] Salama E.R. (2020). Ultrasound-guided bilateral quadratus lumborum block vs. intrathecal morphine for post-operative analgesia after cesarean section: A randomized controlled trial. Korean J. Anesthesiol..

[69] Niklasson B., Arnelo C., Öhman S.G., Segerdahl M., Blanck A. (2015). Oral oxycodone for pain after caesarean section: A randomized comparison with nurse-administered iv morphine in a pragmatic study. Scand. J. Pain.

